# Abnormal iron homeostasis and neurodegeneration

**DOI:** 10.3389/fnagi.2013.00032

**Published:** 2013-07-30

**Authors:** Barry B. Muhoberac, Ruben Vidal

**Affiliations:** ^1^Department of Chemistry and Chemical Biology, Indiana University-Purdue University IndianapolisIndianapolis, IN, USA; ^2^Division Neuropathology, Indiana Alzheimer Disease Center and Department of Pathology and Laboratory Medicine, Indiana University School of MedicineIndianapolis, IN, USA

**Keywords:** neurodegeneration, neuroferritinopathy, ferritin, inclusion bodies, iron, oxidative stress

## Abstract

Abnormal iron metabolism is observed in many neurodegenerative diseases, however, only two have shown dysregulation of brain iron homeostasis as the primary cause of neurodegeneration. Herein, we review one of these - hereditary ferritinopathy (HF) or neuroferritinopathy, which is an autosomal dominant, adult onset degenerative disease caused by mutations in the ferritin light chain (FTL) gene. HF has a clinical phenotype characterized by a progressive movement disorder, behavioral disturbances, and cognitive impairment. The main pathologic findings are cystic cavitation of the basal ganglia, the presence of ferritin inclusion bodies (IBs), and substantial iron deposition. Mutant FTL subunits have altered sequence and length but assemble into soluble 24-mers that are ultrastructurally indistinguishable from those of the wild type. Crystallography shows substantial localized disruption of the normally tiny 4-fold pores between the ferritin subunits because of unraveling of the C-termini into multiple polypeptide conformations. This structural alteration causes attenuated net iron incorporation leading to cellular iron mishandling, ferritin aggregation, and oxidative damage at physiological concentrations of iron and ascorbate. A transgenic murine model parallels several features of HF, including a progressive neurological phenotype, ferritin IB formation, and misregulation of iron metabolism. These studies provide a working hypothesis for the pathogenesis of HF by implicating (1) a loss of normal ferritin function that triggers iron accumulation and overproduction of ferritin polypeptides, and (2) a gain of toxic function through radical production, ferritin aggregation, and oxidative stress. Importantly, the finding that ferritin aggregation can be reversed by iron chelators and oxidative damage can be inhibited by radical trapping may be used for clinical investigation. This work provides new insights into the role of abnormal iron metabolism in neurodegeneration.

## Introduction

Hereditary ferritinopathy (HF) or neuroferritinopathy is an autosomal dominant, adult onset neurodegenerative disease caused by mutations in the ferritin light chain (FTL) gene (Curtis et al., 2001; Vidal et al., 2004a; Mancuso et al., 2005; Ohta et al., 2008; Devos et al., 2009; Kubota et al., 2009). The disease was first reported in members of two families from England and France and was named neuroferritinopathy (Curtis et al., 2001). Sequence analysis of the *FTL* gene in members of the English family disclosed an adenine duplication, which predicts alteration of the C-terminal FTL polypeptide sequence and length (Curtis et al., 2001). Thus, far, six different mutations in exon four of the FTL gene have been reported, all affecting the FTL C-terminus. The clinical phenotype of HF is characterized by a movement disorder, behavioral abnormalities, and cognitive impairment. The brain shows cerebral and cerebellar atrophy and cavitation of the putamen. The main neuropathologic findings are the presence of intranuclear and intracytoplasmic ferritin inclusion bodies (IBs) in glial cells and in some subsets of neurons, and abnormal iron deposition. Molecular level investigations of ferritin containing the mutant subunit reveal functional defects of iron mishandling, ferritin aggregation, and oxidative damage. These processes are linked to a structural defect in the FTL C-terminus leading to cellular dysfunction that can be broadly classified as a loss of normal function and gain of toxic function as discussed below.

## Ferritin structure, iron chemistry, and protein aggregation

**Figure 1:**
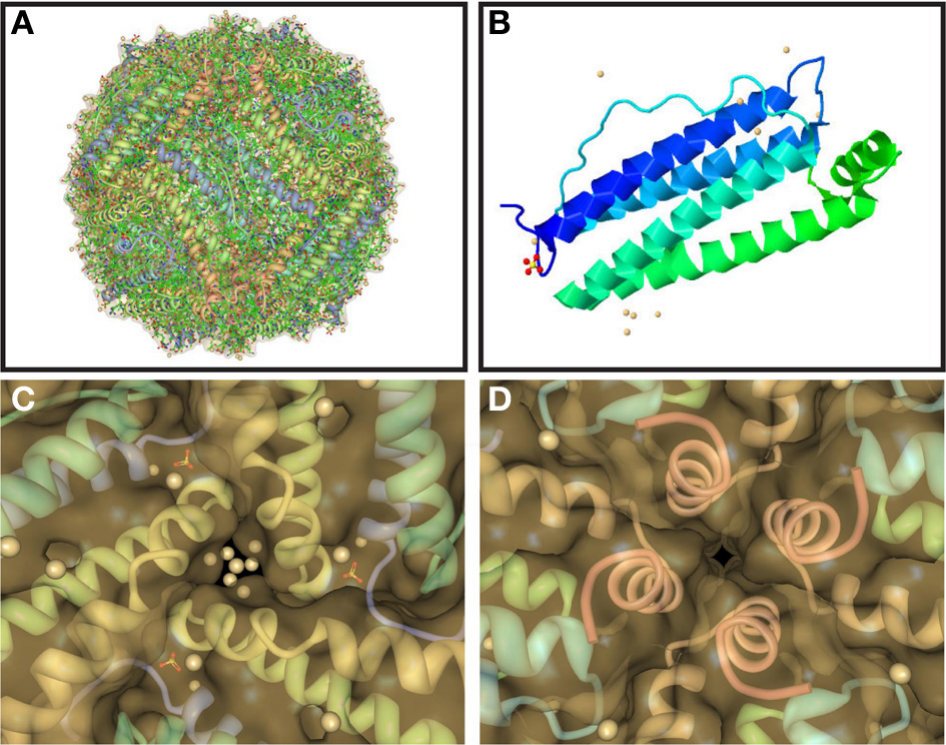
**Structure of ferritin, one of its 24 subunits, and its pores**. Ferritin assembles spontaneously from 24 conformationally equivalent subunits into a hollow spherical shell **(A)** with 2-, 3-, and 4-fold symmetry axes. The subunits **(B)** are each composed of five α-helices with four of them parallel and tightly associated and the fifth (the E-helix) at an angle and pointed inwards. The junctions of the subunits form hydrophilic pores at the 3-fold axes **(C)**, which are implicated as the entry path for iron ions, and hydrophobic pores at the 4-fold axes **(D)**, which are smaller and considered closed, especially to ions. Pores are viewed from the ferritin shell interior with the inwardly pointing E-helices from the 4 subunits clearly visible in **(D)**. Structures were taken from RCSB (code 2FG8).

Ferritin is uniquely suited for its crucial iron sequestration and storage function. Ferritin consists of 24 subunits that can self-assemble into a 480 kDa hollow sphere of ~110 Å outer and ~80 Å inner diameter (Figures 1A, B), which can store up to 4500 atoms of iron as a ferrihydrite biomineral (Vidal et al., 2004b; Crichton and Declercq, 2010). The exterior and interior of the ferritin shell are connected via channels (pores) along 3-fold and 4-fold symmetry axes at subunit junctions. There are eight 3-fold pores that are larger diameter and shorter relative to the six 4-fold pores. The 3-fold pores (Figure 1C), which are hydrophylic, have been implicated as the iron entry pathway in a number of studies, whereas the 4-fold pores (Figure 1D), which are hydrophobic, are smaller and considered essentially closed, especially to ions. Human ferritin is usually heteropolymeric with the 24-mer formed from two conformationally equivalent subunits with slightly different masses and 54% sequence identity. The two subunits are folded from the ferritin heavy chain (FTH1) and light chain (FTL) polypeptides with the former longer by 8 amino acids. Both subunits consist of four parallel α-helices (A–D) of ~45 Å length and a shorter, carboxy terminal α-helix (E) at a 60° angle to the parallel helix bundle pointing into the interior of the shell (Figure 1B) (Crichton and Declercq, 2010). The 4-fold pores are formed from four hydrophobically-associated E-helices donated from four different subunits (Figure 1D), while 3-fold pores are formed from C- and D-helices from three subunits (Figure 1C). The FTH1 subunit contains the ferroxidase site in the interior of the parallel helix bundle, which converts Fe^2+^ to Fe^3+^ in the presence of O_2_ on the pathway to biomineralization. Iron then migrates to the ferrihydrite nucleation site on the interior surface of the FTL subunit to complete biomineral formation. Interestingly, each organ fine tunes the ratio of FTH1 to FTL subunits, which can vary substantially, for optimal physiological function. No homopolymers of the FTH1 subunit have been reported, but homopolymeric ferritin formed from 24 FTL subunits is capable of a slow rate of iron sequestration and storage. The FTL subunit is more stable than the FTH1 subunit toward increasing temperature and denaturants, and when incorporated into the heteropolymer stabilizes it and hinders iron-induced aggregation (Santambrogio et al., 1992, 1993).

Properly functioning ferritin of the appropriate cellular concentration is essential for iron homeostasis, and a number of neurodegenerative diseases have links with misregulation of iron (Zecca et al., 2004; Berg and Youdim, 2006). The normal cellular response to abundant iron is to decrease the synthesis of the transferrin receptor, which transports iron into the cells, and to increase ferritin synthesis for appropriate iron sequestration and storage. During iron deficiency the synthesis of ferritin is inhibited in part by an increase in Iron Responsive Protein (IRP) binding to the Iron Responsive element (IRE) on target mRNAs. Clearly, iron must be made available to form the essential catalytic centers of neuroenzymes, e.g., tryptophan hydroxylase, which is required for serotonin synthesis, and tyrosine hydroxylase leading to dopamine. However, local iron concentration and its ligation (coordination) and oxidation state must all be carefully regulated to prevent cellular damage. Indeed, the chemistry that occurs at the iron can vary substantially depending on iron ligation, which could be provided by protein donors (e.g., certain amino acid side chains) or small molecules normally available in cells. Much cellular chemistry is driven by the process of redox change, and cells have available the redox drivers of reductants such as NADPH, glutathione, and ascorbate, as well as oxygen. Hydrogen peroxide and superoxide are generated in cells as part of several routine metabolic redox processes as well as by non-enzymatic reduced iron, and although they can deactivate a few enzymes, they are not particularly damaging at appropriate levels (Zecca et al., 2004). In fact, hydrogen peroxide has recently been characterized as a neuromodulator in striatal dopamine release (Rice, 2011). However, improperly coordinated iron has the potential to convert hydrogen peroxide and superoxide into the highly toxic hydroxyl radical, which is extraordinarily reactive. This radical indiscriminately attacks proteins, lipids, and DNA causing protein oxidation, fragmentation, and covalent crosslinking leading to their loss of function. Hydroxyl radical production occurs through redox change in metal-centered Fenton- and Haber-Weiss- type reactions, and when iron is coordinated by less than 6 strongly-bound ligands this conversion is facilitated (Graf et al., 1984). Hydrogen peroxide, superoxide, and the hydroxyl radical are generally termed reactive oxygen species (ROS), and cells produce enzymes (e.g., glutathione peroxidase, catalase, and superoxide dismutase) and small molecules (glutathione) to help control their levels (Murphy et al., 2011). The ability of ferritin to convert Fe^2+^ to Fe^3+^ and store it internally as a non-reactive biomineral can be considered a “detoxification” function by removing it from potentially inappropriate ligation and reactions. Although usually considered as antioxidants, glutathione and ascorbate have concentrations in the brain that are sufficiently high enough to provide reducing equivalents that can generate ROS and thus lead to cellular damage in the presence of improperly coordinated iron. Along these lines, during iron excess the likelihood of inappropriate iron coordination by normally available small molecule cellular constituents, which are not part of proteins and usually not problematic, is increased potentially causing the generation of ROS beyond basal levels and protein oxidation.

Proper disposal or reconstitution of damaged or misfolded proteins to avoid aggregation is an ongoing necessary cellular function, and protein aggregation is strongly linked with neurodegenerative diseases (Lansbury and Lashuel, 2006). Cells devote significant resources energetically through specialized protein synthesis and transport toward the disposal or reconstitution of oxidized or misfolded proteins, which are generally prone to aggregate formation. The aggregation process is dependent upon a variety of factors besides the identity and extent of abnormality of the primary protein involved such as its concentration, cellular crowding by other proteins, and the presence of small molecules and metal ions. However, the aggregation process is generally thought to follow one of two paths. The misfolded protein first forms dimers, trimers, and oligomers with a structural rearrangement, and then forms highly ordered and symmetric structures, e.g., fibrils in Alzheimer disease. Alternately the aggregation process may involve less ordered clumping or packing together of proteins that leads to various tangles or IBs. Often the most toxic forms are the smaller oligomers and poorly ordered aggregates, and there is substantial evidence for this with the larger structures considered neuroprotective. However, the larger, microscopically visible IBs may not be inert cellular end products, but influence metabolism, transport and structure, perhaps even through causing detrimental mechanical crowding.

Transition metal ions such as iron, copper and zinc are often found at elevated levels in neurodegenerative diseases and are intimately connected with protein aggregation, misfolding and cellular dysfunction (Ayton et al., 2012). For example, the addition of iron to solutions of α-synuclein or amyloid β enhances the rate of aggregation and/or fibril formation even to the extent of influencing the structure of the aggregates formed, and elevated iron is associated with the pathology of these disorders in both animal models and humans. Metal ion binding can be causal to aggregation and misfolding, or be advantageous after they occur. However, metal ion-induced aggregated and misfolded proteins, while problematic for cellular processes and elimination, could foster an additional cellular dysfunction through providing binding sites that improperly ligate (coordinate) iron and thus generate ROS. The resulting oxidative damage itself can enhance protein aggregation. In Huntington disease IBs form iron-dependent centers of oxidative damage causing alterations in the cellular morphology of their surroundings (Firdaus et al., 2006). Cells devote resources to respond to damaged or aggregated proteins by synthesis of enzymes to repair or degrade them. As will be discussed below, mutant ferritin is intimately associated with iron mishandling, aggregation, and radical damage of proteins, which can be traced directly to a protein structural abnormality resulting from its disordered C-terminal helices and unstructured 4-fold pores (Baraibar et al., 2010).

## Clinical presentation, genetics, and pathology of HF

The neurodegenerative disease HF has been reported in members of Caucasian and Japanese families, being inherited in an autosomal dominant pattern. Linkage analysis, performed on members of an English family, linked the disease to a locus on chromosome 19q13.3, which contains the *FTL* gene. Affected individuals from this family were found to have an adenine insertion at position 460–461 of the *FTL* gene (*c.460dupA*) that was predicted to alter the carboxyl-terminal region of the protein (Curtis et al., 2001). Five additional mutations have been reported, all in exon 4 of the *FTL* gene, which consists of 4 exons and 3 introns. In all cases, the mutations (Table 1) affect the E-helix of the FTL polypeptide by altering both the C-terminal sequence and extending its length. In addition to these, two more cases of HF have been described. The first case was diagnosed pathologically and no genetic data is available (Schröder, 2005), while the second case was described as an individual with a missense mutation (A96T) in FTL (Maciel et al., 2005). In the later, it remains to be seen whether this case reflects a bigger spectrum of the disease or a different condition since the mother of the proband (also a carrier of the A96T mutation who displayed similar MRI findings) was asymptomatic (lack of autosomal dominant transmission), and the patient did not have significant involvement of the putamen, thalamus, and substantia nigra.

**Table 1:**
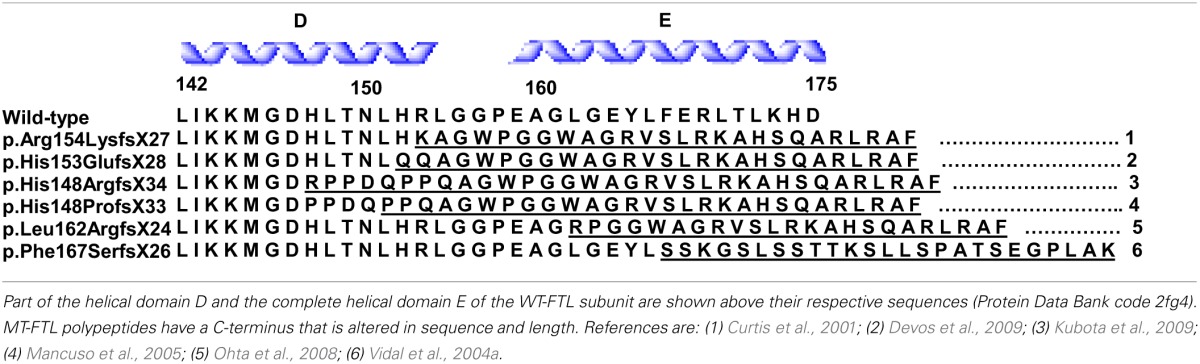
**Sequence alignment of WT- and MT-FTL polypeptides starting at residue 142**.

Part of the helical domain D and the complete helical domain E of the WT-FTL subunit are shown above their respective sequences (Protein Data Bank code 2fg4). MT-FTL polypeptides have a C-terminus that is altered in sequence and length. References are: (1) Curtis et al., 2001; (2) Devos et al., 2009; (3) Kubota et al., 2009; (4) Mancuso et al., 2005; (5) Ohta et al., 2008; (6) Vidal et al., 2004a.

**Figure 2:**
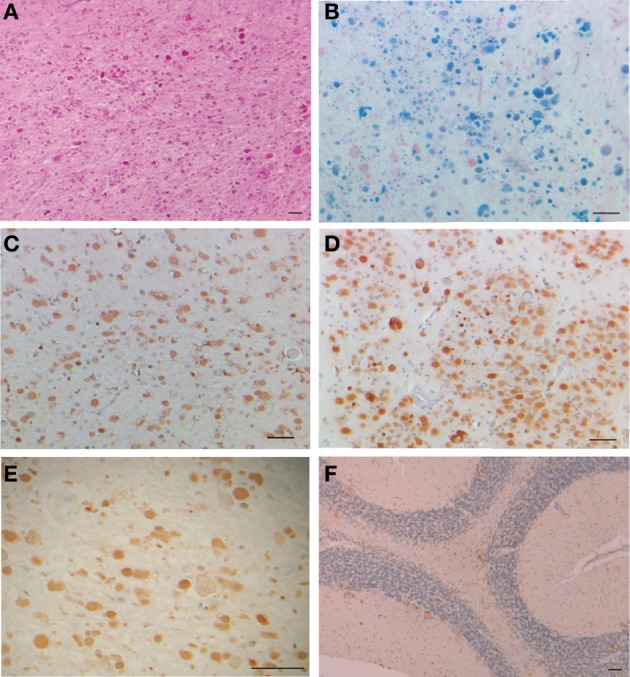
**Inclusion bodies, iron deposits, and immunohistochemistry from a patient with hereditary ferritinopathy**. Sections of putamen show numerous ferritin IBs of various sizes **(A–E)**, which are ubiquitinated **(E)**. Ferritin IBs were also present in neurons and glial cells of the cerebellum **(F)**. Hematoxylin and eosin **(A)**; Perls' Prusian blue method for iron **(B)**; and immunohistochemistry using antibodies against mutant FTL **(C)**, wild-type FTL **(D)**, ubiquitin **(E)**, and FTH1 **(F)**. Scale bars: **(A–F)**, 50 μm.

Clinically, HF presents as a movement disorder syndrome similar to Huntington disease or Parkinson disease. The disease may present with tremor, cerebellar signs, Parkinsonism, psychiatric problems, abnormal involuntary movements (dystonia, chorea), pyramidal syndrome, pseudo-bulbar symptoms, and cognitive deficit (Caparros-Lefebvre et al., 1997; Curtis et al., 2001; Vidal et al., 2004a; Mancuso et al., 2005; Chinnery et al., 2007; McNeill et al., 2008; Ohta et al., 2008; Devos et al., 2009; Kubota et al., 2009; Ory-Magne et al., 2009). The clinical presentation of HF has been reported to differ both within and between families (Ory-Magne et al., 2009), usually becoming evident in the third to fifth decade of life (depending on the specific mutation) and progressing unalleviated thereafter. Neuroimaging studies show abnormal signals in the globus pallidus and putamen, and cavitation of the putamen, while serum ferritin levels were reported to be decreased in some patients (Curtis et al., 2001; Ory-Magne et al., 2009). Neuropathological data is available for individuals with the *c.442dupC, c.460dupA*, and *c.497_498dupTC* mutations (Curtis et al., 2001; Vidal et al., 2004a; Mancuso et al., 2005). Examination of the brain shows mild cerebral and cerebellar atrophy as well as cavitations of the putamen. The main neuropathologic findings (Figure 2) are the presence of intranuclear and intracytoplasmic ferritin IBs in glial cells and in some subsets of neurons, substantial iron deposition, and mild to moderate nerve cell loss and gliosis. Glial cells containing IBs are mostly found in the caudate nucleus, putamen, and globus pallidus. These areas show severe nerve cell loss, extracellular ferritin deposits, and loss of neuropil. In the cerebral cortex, IBs are seen in perineuronal cells and in perivascular glia. The presence of IBs in neurons is clearly observed in the putamen, globus pallidus and thalamus, and in cerebellar granule cells and in Purkinje cells (Vidal et al., 2004a). Intranuclear inclusions are large enough to almost completely occupy the nucleus mechanically forcing chromatin against the nuclear membrane. IBs can be seen as homogenous, eosinophilic bodies, which can be labeled by antibodies against FTL and FTH1 polypeptides of ferritin and by antibodies specific for the mutant FTL polypeptide. Inclusions also contain Fe^2+^ and Fe^3+^, as determined by Turnbull blue and by Perls' or Prussian blue, respectively. By transmission electron microscopy (TEM), nuclear IBs are seen as composed of small (~100 Å) granular electron-dense particles that resemble ferritin and occupy a large portion of the nucleoplasm. IBs have been reported in the skin, kidney, liver, and muscle in affected individuals from French and American families (Vidal et al., 2004a; Mancuso et al., 2005). The presence of ferritin IBs in skin or muscle biopsies may help in the diagnosis of the disease.

## Functional abnormalities in mutant-containing ferritin

The isolation and biochemical analysis of IBs from individuals with HF identified wild-type FTL, FTH1 and mutant FTL polypeptides as the main components of IBs (Vidal et al., 2004a). Although ferritin is generally isolated as a heteropolymer of FTH1 and FTL subunits, initial structural and functional studies focused on the biological significance of the mutant as a 24-mer in its homopolymeric form. Thus, recombinant wild type (WT)- and mutant (MT)-FTL (p.Phe167SerfsX26) polypeptides were separately expressed in *E. coli*, reconstituted with all iron removed, and analyzed. Both polypeptides were soluble and assembled as 24-mer homopolymers by size exclusion chromatography (SEC). TEM analysis showed that the ferritin particle had a spherical shape and size (outer diameter ~110 Å) similar to that of human ferritin (Baraibar et al., 2008). When WT-FTL apoferritin homopolymers (1 μM) were iron-loaded following a routine procedure by aerobic incubation with up to 4500 iron atoms per 24-mer of ferrous ammonium sulfate for 2 h, no precipitation was found. However, precipitation of the Mt-FTL homopolymer was observed to begin when the number of iron atoms was higher than ~1500 iron atoms per ferritin 24-mer. By monitoring direct iron incorporation by native PAGE followed by Prussian blue stain, it was observed that at moderate iron loading of up to ~1000 iron atoms per ferritin 24-mer and 2 h incubation, WT- and MT-apoferritin homopolymers incorporated very similar amounts of iron, which implies a degree of functionality for both. However, at higher iron to ferritin ratios, WT-FTL homopolymers continued incorporating iron, whereas incorporation by MT-FTL homopolymers dropped precipitously. The change in absorbance at 310 nm measured during the first 500 s after mixing iron and ferritin in a 1000 to 1 ratio was substantially larger for WT-FTL vs. MT-FTL homopolymers, uncovering a clear difference in iron handling between mutant and wild type ferritin at early times after mixing (Baraibar et al., 2010). When in separate solutions, both WT- and MT-FTL homopolymers showed significant ability to incorporate iron, but when in direct competition (in the same solution), there was complete absence of iron incorporation by MT-FTL. No precipitation was noted, which highlights the importance of direct iron mishandling by mutant ferritin without the effects of the iron-induced precipitation. The direct role of iron in the precipitation of MT-FTL homopolymers was further emphasized by using the iron chelator deferoxamine (DFX). Importantly, it was observed that greater than 50% of mutant homopolymers that were precipitated by the addition of iron (3500 iron atoms per 24-mer) were re-solubilized by incubation with DFX (Baraibar et al., 2008), highlighting chelation as a major *in vitro* modulator of MT-FTL aggregation, which is an important marker for HF *in vivo*.

These homopolymeric studies were followed by examination of ferritin heteropolymers reconstituted with an equal number of mutant and wild type subunits in each 24-mer (Muhoberac et al., 2011). The intermediate mobility on SDS-PAGE and overlap in SEC profiles were indicative of appropriate co-assembly. It was found that both forms of heterepolymers containing the MT-FTL subunit (MT-FTL/FTH1, MT-FTL/WT-FTL) and again MT-FTL homopolymers themselves were significantly more susceptible to iron-loading induced precipitation than either the WT-FTL/FTH1 heterepolymer or the WT-FTL homopolymer, when analyzed by the same above-mentioned routine procedure. Thus, reconstitution of mutant with either wild type does not rescue ferritin from the iron-induced aggregative behavior (Muhoberac et al., 2011). Furthermore, a direct measure of iron incorporation using native PAGE and Prussian blue stain showed that WT-FTL/FTH1 was twice as capable of incorporating iron than MT-FTL/FTH1 heteropolymers. Importantly, some iron was still incorporated with mutant-containing ferritin suggesting some level of functionality.

Recently, oxidation of MT-FTL was observed to occur both *in vitro* and *in vivo* in individuals with HF (Baraibar et al., 2012). Incubation of MT-FTL homopolymers with physiological concentrations of iron and ascorbate resulted in shell structural disruption and polypeptide cleavage not found under the same conditions with WT-FTL. Along with the ~21 kDa FTL polypeptide were found fragments of ~ 6 and ~14 K Da, as well as one of ~27 kDa suggesting covalent crosslinking. Mutant ferritin also underwent a 2.5-fold increase in carbonyl group formation over wild type. Polypeptide cleavage and shell disruption was completely inhibited by addition of the radical trap 5,5-dimethyl-1-pyrroline N-oxide, indicating an enhanced propensity of MT-FTL 24-mers toward free radical-induced, oxidative damage *in vitro*. Importantly, IBs from a patient with HF exhibited extensive carbonylation together with an isolatable C-terminal MT-FTL fragment of ~14 kDa, which are both indicative of *in vivo* oxidative ferritin damage. These data point toward a connection between oxidative damage, mutant ferritin, and HF, and suggest that radical scavengers (i.e., more generally antioxidants) and iron chelators have the potential to be therapeutic agents for treatment of HF.

## E-helix disruption and enhanced aggregation in mutant-containing ferritin

**Figure 3:**
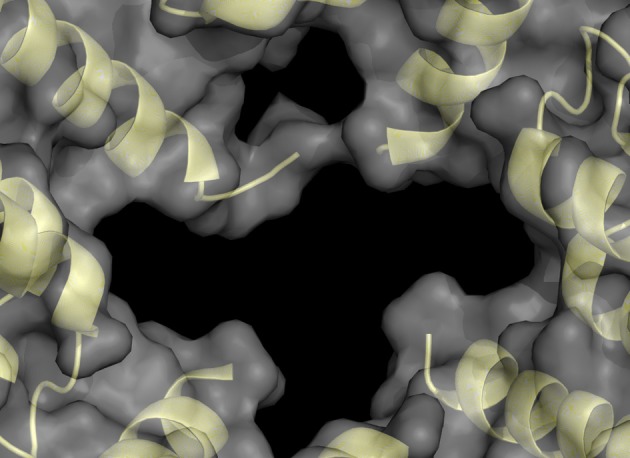
**Structural disruption of the 4-fold pores of ferritin caused by the C-terminal mutation**. Each wild type 4-fold pore is formed from four tightly associated E-helices, one donated from each subunit, as seen in Figure 1D. With the mutant, the E-helices are unraveled causing significant disruption of the 4-fold pores and providing extended and disordered C-termini, which are not visible by X-ray crystallography. This MT-FTL 4-fold pore is an example of several, which vary somewhat in disruption details, and is viewed from the ferritin shell interior. The structure was taken from RCSB (code 3HX5).

**Figure 4:**
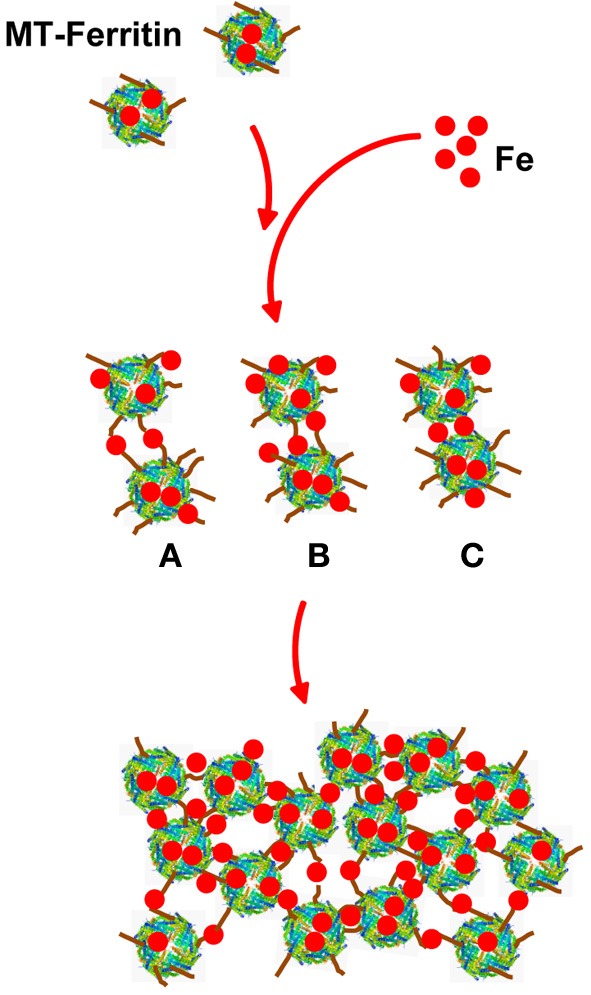
**Model of iron-induced aggregation of ferritin containing the C-terminal mutation**. Unraveled MT-FTL C-terminal E-helices (brown lines) can extend a substantial distance from the ferritin shell surface into the solvent providing a number of groups that can coordinate iron. Addition of iron to a solution of MT-FTL-containing ferritin initiates bridging of C-termini by iron (or iron nucleation complexes) reducing their translational motion. Cross-linking may occur between two separate ferritin 24-mers through iron bridges between C-termini **(A)**, between a C-terminus and surface iron-binding amino acid side chain **(B)**, and/or eventually through surface amino acids that bind iron on both 24-mers **(C)** forming ferritin aggregates [adapted from Baraibar et al. (2008)].

The structure of the spherical protein shell is maintained in mutant ferritin as was seen in the crystallographic structures of the MT-FTL (p.Phe167SerfsX26) homopolymers (Baraibar et al., 2010). However, a close up examination of the 4-fold pores showed remarkable disruption of the MT-FTL C-terminal helices making the pores unstable and leaky (Figure 3 vs. Figure 1D). Because as many as the last 26 amino acids of MT-FTL remained unaccounted for crystallographically, mutant C-termini may extended and reach as far as the diameter of the ferritin shell itself. The C-terminal sequence of the mutant contains a number of groups known to bind iron, e.g., the C-terminal carboxylate, glutamate, tyrosinate and the hydroxyl groups of several serines and threonines. Considering the sensitivity of ferritin-containing MT-FTL to *in vitro* and *in vivo* (see below) iron-induced aggregation, a model was proposed in which iron binds to the unraveled and extended mutant C-termini on two different ferritin shells bridging them and initiating a gradual aggregation of ferritin and iron into a precipitate (Figure 4). Bridging is not necessarily restricted to C-termini and may become more general, e.g., between a C-terminal group and a surface amino acid which both have affinity for iron (Baraibar et al., 2008, 2010). This model has potential additional complexities because of (1) the very strong dependence of the strength of iron binding to certain groups on its redox state and (2) the existence of small iron hydroxide nucleation centers that may form in solution without the presence of protein.

The role of the mutant C-terminus and its protrusion above the protein shell in the iron-induced aggregation process was characterized further by comparing the iron loading of apoferritin homopolymers composed of WT-FTL, MT-FTL and a C-terminally truncated FTL polypeptide (p.S167X). The assembly status of the truncated FTL polypeptide as a homopolymer (24-mer) was verified by SEC and gel electrophoresis. In contrast to MT-FTL homopolymers, which began to precipitate at ~1500 iron atoms per 24-mer, both the WT-FTL and truncated FTL homopolymers remained in solution up to a ratio of 4000 to 1. Thus, removal of the mutant portion of the C-terminus prevented iron-induced precipitation reinforcing the importance of the interaction between iron and the disordered C-terminus in the aggregation process (Baraibar et al., 2010).

## Iron chelation and radical trapping in animal and cellular models of HF

**Figure 5:**
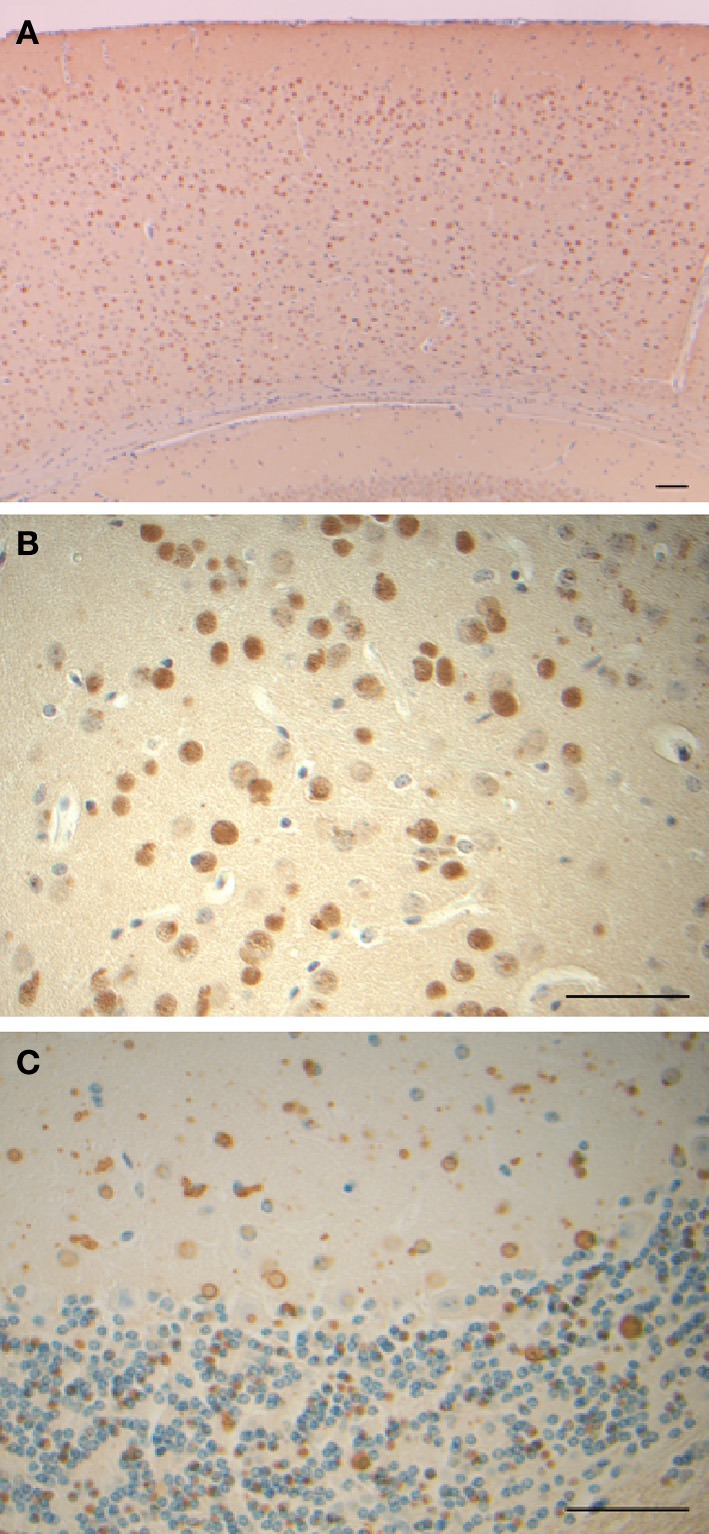
**Inclusion bodies and immunohistochemistry from a transgenic mouse model of hereditary ferritinopathy**. Sections of cerebral cortex **(A)**, globus pallidus **(B)**, and cerebellum **(C)** of FTL-Tg mice show the presence of numerous ferritin IBs. Sections were from a 9 month old homozygous male **(A,B)** and an 11 month old heterozygous male **(C)**. Immunohistochemistry was performed using antibodies against the N-terminus of wild-type and mutant FTL **(A–C)**. Scale bars: **(A–C)**, 50 μm.

A transgenic animal model of HF (FTL-Tg) was generated in order to increase our understanding of the effects of MT-FTL on brain iron metabolism and ferritin expression and disposition. A human *FTL* cDNA carrying a thymidine and cytidine insertion at position 498 (*c.497_498dupTC*) was expressed in mouse, leading to iron mishandling, ferritin accumulation, and oxidative stress (Vidal et al., 2008). Expression of the transgene caused the formation of nuclear and cytoplasmic ferritin IBs in glia and neurons throughout the CNS (Figure 5), as well as in cells of other organ systems. The size and number of nuclear inclusions increased with age becoming large enough to cause mechanical crowding and displacement of chromatin, as was found in HF patients (Vidal et al., 2004a). FTL-Tg mice had a progressive neurological phenotype, a significant decrease in motor performance, and a shorter lifespan. These mutant mice showed an increase in brain iron and altered levels of associate proteins. Cytoplasmic FTL and FTH1 polypeptides increased and the transferrin receptor level decreased, as would be the expected in response to excess iron. Ubiquinated proteins and portions of the proteosome (20S, 11S, and 19S) accumulated in the IBs, implying cellular recognition of the presence of abnormal protein aggregates. FTL-Tg mice showed accumulation of oxidative DNA damage in brain mitochondria, but no significant damage to nuclear DNA (Deng et al., 2010). Furthermore, markers for oxidative stress such as protein carbonyl formation, nitrone-protein adducts, and lipid peroxidation, were found in the brain indicative of cellular damage by ROS (Barbeito et al., 2009).

Ferritin levels in primary cultures of astrocytes from the cerebral cortex of FTL-Tg mice respond dramatically to exposure to iron and chelators. Cell treatment with 50 uM ferric ammonium citrate (FAC) caused a switch of MT-FTL ferritin from the detergent-soluble to the detergent-insoluble fraction, strongly supporting a role for iron in the formation of IBs. After removal of FAC solution, addition of 50 uM of the lipophilic iron chelator 1,10-phenanthroline to the FAC-treated cells led to a large reduction in detergent-insoluble ferritin and the reappearance of ferritin in the detergent-soluble fraction. Phenanthroline is a freely cell-permeable chelator and was chosen for the study over the weakly cell-permeable chelator deferroxamine (Baraibar et al., 2008). These studies show that IB formation is strongly dependent on iron levels and can be reversed by using iron chelators *in vivo*, which supports chelation therapy as a potential treatment to inhibit aggregation and reduce IB formation in HF.

Primary cultures of human skin fibroblasts from patients with HF were also used to characterize the effects of MT-FTL on cellular iron metabolism (Barbeito et al., 2010). These cells exhibited iron mishandling, ferritin accumulation, and evidence of oxidative stress, paralleling the dysfunction seen in both patients with HF and the mouse model. Mutant fibroblasts showed a significant increase in the level of total iron content under basal metabolic conditions when compared to wild type fibroblasts, but interestingly, without a significant difference in the level of the liable iron pool, which is the iron more readily available for metabolism. Cellular levels of MT-FTL, WT-FTL, and FTH1 polypeptides were all substantially increased in HF vs. wild type fibroblasts. IRE-IRP binding was reduced in HF fibroblasts consistent with the observed enhanced ferritin and decreased transferrin receptor-1 synthesis, and broadly consistent with higher total iron levels in HF fibroblasts. Significant higher levels of ROS were found in HF vs. non-HF fibroblasts (Barbeito et al., 2010), which supports antioxidant therapy as a potential treatment for HF.

## Abnormal ferritin function caused by the mutant polypeptide

**Figure 6:**
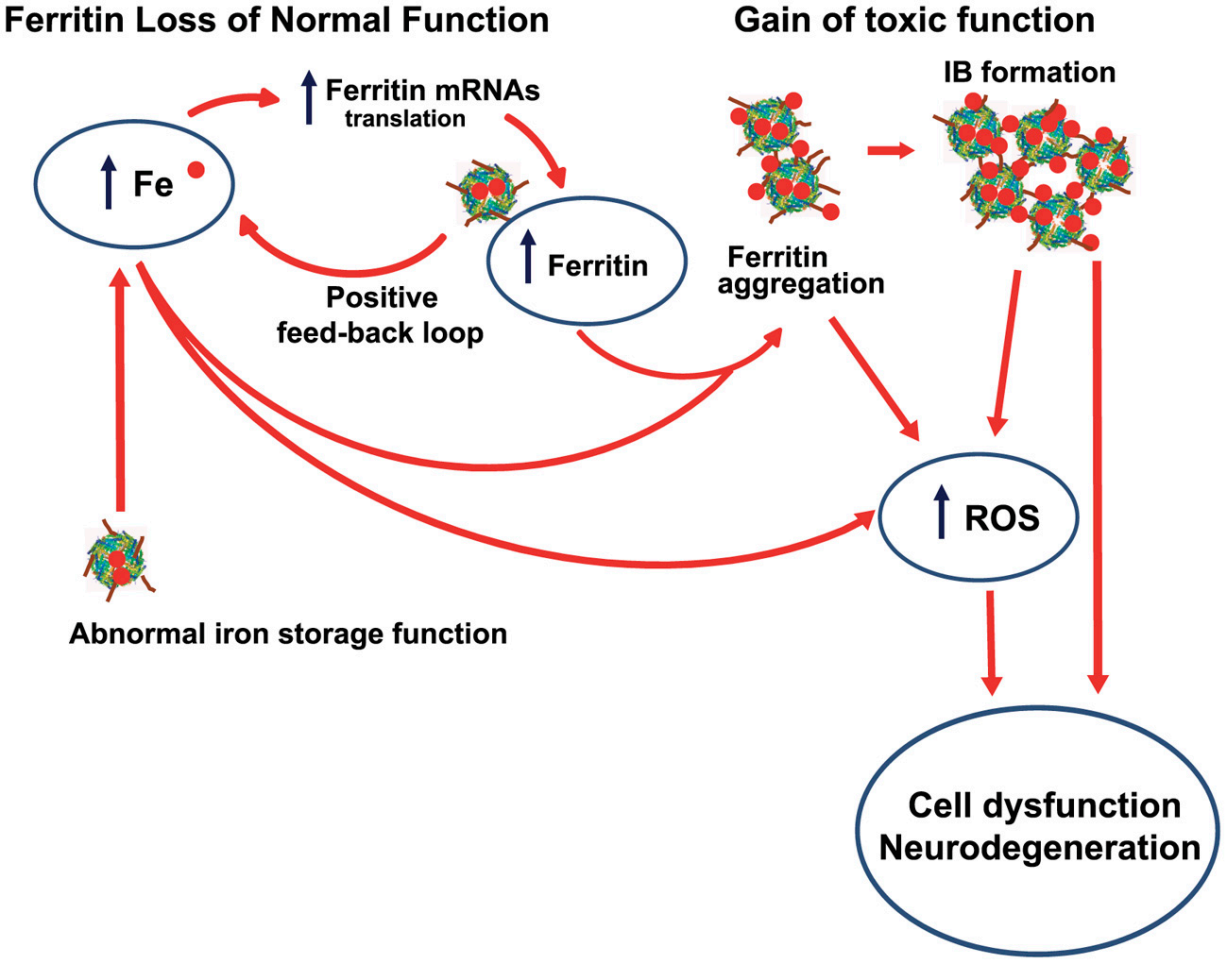
**Interrelationship between iron accumulation, ROS generation, inclusion body formation, and neurodegeneration in hereditary ferritinopathy**. The pathogenesis of HF is consistent with (1) a loss of normal ferritin function through a decrease in iron incorporation into ferritin that triggers intracellular iron accumulation and overproduction of ferritin polypeptides (a positive feedback loop) and (2) a gain of toxic function through radical production, ferritin aggregation, and oxidative stress. ROS can form at improperly ligate iron bound to small ferritin aggregates and IBs, as well as to other cellular constituents (e.g., small molecules) because of elevated iron as represented by the lowest curved arrow in figure. The most toxic ROS is the hydroxyl radical that indiscriminately attack proteins, lipids and DNA causing extensive cellular damage.

Hereditary ferritinopathy provides a direct link between abnormal iron homeostasis and neurodegeneration. The pronounced cellular dysfunction in the pathogenesis of HF centers on three observable and well-characterized cellular abnormalities - iron accumulation, IB formation, and protein oxidation - as described in this review. All of these abnormalities involve a specific molecular-level defect of the MT-FTL C-terminal sequence causing (1) 4-fold pore disruption with reduced ability to store and sequester iron, and (2) unraveling and extension of the C-terminus causing iron-induced aggregation of ferritin. Both (1) and (2) contribute to increasing iron levels leading to the misdirected cellular response of synthesis of more ferritin to sequester iron, which in turn, creates a destructive positive feedback loop that accumulates ferritin and taxes cellular resources. Handling increasing levels of improperly sequestered and stored iron is problematic. Improperly ligated iron, whether this occurs on smaller ferritin aggregates, in IBs, or even through coordination by small molecule cellular constituents, produces ROS leading to protein oxidation, as is evident in the *in vitro* and *in vivo* studies reviewed here. Increasing levels of iron also enhance the aggregation of MT-FTL-containing ferritin, further exacerbating the situation. IBs contain both Fe^2+^ and Fe^3+^, which suggests that ROS could be generated within them. Although HF is an autosomal dominant disease, the age at onset of the disease is not in early childhood, suggesting that the cells are apparently able to handle the many insults initially, but later succumb to cumulative damage. Taken together, a working hypothesis (Figure 6) for the role of MT-FTL in the pathogenesis of HF consistent with the results presented here implies both (1) a loss of normal ferritin function (decreased iron incorporation) that triggers intracellular iron accumulation and overproduction of ferritin polypeptides, and (2) gain of toxic function through radical production, ferritin aggregation, and oxidative stress. The concept of loss of normal function and gain of toxic function may have applicability to the understanding of other disease processes, especially when considering interactions with transition metal ions and available cellular reductants. More generally, a protein mutation or conformational change that leads to loss of normal function may also allow the ligation of transition metals to abnormal protein binding sites with the two major consequences of ROS generation and protein aggregation, which are both a gain of toxic function. It should be noted that zinc, although not redox active, can induce substantial protein aggregation, and may thus play an important role (Ayton et al., 2012). The loss of normal function could lead to a positive feedback loop in compensating cellular protein synthesis producing more abnormal protein metal binding sites, and could be further complicated if the protein in question normally handles transition metal ion transport or removal. It is somewhat ironic that the major iron storage protein ferritin acquires through mutation both the inability to properly handle iron and an enhanced aggregative sensitivity toward the metal that it is designed to keep in homeostasis.

## Outlook for treatment

Currently there is no effective treatment for HF. Therapies aimed at decreasing iron levels or inhibiting ferritin synthesis would appear to be indicated in view of the pivotal roles played by ferritin and iron in cellular metabolism. Decreasing iron levels toward normal or eliminating mutant FTL polypeptide synthesis in HF patients by the more direct approach of gene therapy using viral transfer and expression that could influence iron or ferritin levels would be time consuming, costly to develop, and with some uncertainty of efficacy. Decreasing iron levels toward normal with appropriately designed chelators would reduce ROS production, pathological iron-induced aggregation, and IB formation. However, use of the iron chelators desferrioxamine and deferiprone (as well as venesection) was reported to cause profound and refractory iron depletion without clinical benefits (Chinnery et al., 2007). Although this initially may sound discouraging, the relatively limited number and variety of chelators examined until now as treatment for HF patients should be carefully considered. Chelators are characterized by a large number of molecular properties that need to be optimized to match both the complexities of the cellular system and the disease being treated. The choice of a chelator (and in the longer term its discovery and design) is difficult because optimization of one particularly molecular property to the cellular system and disease may adversely affect the optimization of another. For example, adding a particular group to the skeleton of a chelator to optimize lipid- vs. aqueous-solubility may adversely affect its redox properties or binding strength. Indeed, there are a number of considerations to chelator efficacy beyond facile blood-brain barrier penetration and increasing the binding strength as follows: (1) The chelator must selectively bind and release iron and not interfere significantly with the concentration, transport, and distribution of other metals. (2) Iron binding and removal must occur in a manner as not to sequester iron from functioning enzymes that require it for activity. (3) The chelator and iron-chelator complex must be hydrophobic enough to permeate multiple membrane barriers to redistribute iron for eventual excretion. (4) The iron-chelator complex should have redox and coordination properties such that it does not itself serve as a source of ROS using available oxygen and reducing equivalents in the brain. This list of considerations and the fine tuning of chelator molecular properties that it implies stand in contrast to the two chelators already explored to treat HF both in number and molecular composition. More generally this list is in contrast to the few chelators currently approved by the FDA for iron-overload therapy implying that chelation therapy for HF remains mainly unexplored.

Although additional *in vitro* characterization of mutant ferritin in combination with computational approaches may eventually prove useful in defining compounds with optimal molecular properties for drug candidates for HF therapy, it is important to consider application of currently available *in vivo* approaches employing existing animal and cellular models. One approach would be to develop a screening procedure using fibroblasts or astrocytes from HF patients against a small molecule library akin to the NIH Molecular Libraries Program or other similar programs. Whole animal studies using the mouse model would follow. In the best case scenario, the drug candidates would be the same or similar to an approved FDA drug. A more focused approach would perhaps be to screen approved drugs known to have iron deficiency as a side effect.

Although the preceding discussion targets the ability of chelators to remove and redistribute iron and to resolubilize or prevent ferritin aggregation, as was demonstrated to be operational both *in vitro* and *in vivo* by examples in this review, inhibition of oxidative damage by a free radical scavenger was also described. Damage by ROS could be reduced by removal of excess iron, but could also be reduced by the presence of radical scavengers. This points to the investigation of combined drug therapy for HF patients in the form of administering an optimal iron chelator simultaneously with a powerful antioxidant known to easily cross the blood brain barrier. The combined therapy as outlined here, especially if initiated early, may be more successful, not only for HF, but also for other neurodegenerative diseases characterized by brain iron deposition.

### Conflict of interest statement

The authors declare that the research was conducted in the absence of any commercial or financial relationships that could be construed as a potential conflict of interest.
